# BPTF inhibits NK cell activity and the abundance of natural cytotoxicity receptor co-ligands

**DOI:** 10.18632/oncotarget.17834

**Published:** 2017-05-12

**Authors:** Kimberly Mayes, Zeinab Elsayed, Aiman Alhazmi, Michael Waters, Suehyb G. Alkhatib, Mark Roberts, Carolyn Song, Kristen Peterson, Vivian Chan, Nikhil Ailaney, Pumoli Malapati, Tana Blevins, Berislav Lisnić, Catherine I. Dumur, Joseph W. Landry

**Affiliations:** ^1^ The Department of Human and Molecular Genetics, Virginia Institute of Molecular Medicine, Massey Cancer Center, Virginia Commonwealth University, Richmond, Virginia 23298, USA; ^2^ The Department of Biochemistry, Virginia Commonwealth University, Richmond, Virginia 23298, USA; ^3^ The Department of Pathology, Virginia Commonwealth University, Richmond, Virginia 23298, USA; ^4^ The Center for Proteomics and Department for Histology and Embryology, University of Rijeka, Faculty of Medicine, 51000 Rijeka, Croatia

**Keywords:** BPTF, chromatin remodeling, antitumor immunity, NK cell, heparanase

## Abstract

Using syngeneic BALB/c mouse breast cancer models, we show that the chromatin remodeling subunit bromodomain PHD finger transcription factor (BPTF) suppresses natural killer (NK) cell antitumor activity in the tumor microenvironment (TME). In culture, BPTF suppresses direct natural cytotoxicity receptor (NCR) mediated NK cell cytolytic activity to mouse and human cancer cell lines, demonstrating conserved functions. Blocking mouse NCR1 *in vivo* rescues BPTF KD tumor weights, demonstrating its importance for the control of tumor growth. We discovered that BPTF occupies heparanase (*Hpse*) regulatory elements, activating its expression. Increased heparanase activity results in reduced cell surface abundance of the NCR co-ligands: heparan sulfate proteoglycans (HSPGs). Using gain and loss of function approaches we show that elevated heparanase levels suppress NK cell cytolytic activity to tumor cells in culture. These results suggest that BPTF activates heparanase expression, which in turn reduces cell surface HSPGs and NCR co-ligands, inhibiting NK cell activity. Furthermore, gene expression data from human breast cancer tumors shows that elevated *BPTF* expression correlates with reduced antitumor immune cell signatures, supporting conserved roles for BPTF in suppressing antitumor immunity. Conditional BPTF depletion in established mouse breast tumors enhances antitumor immunity, suggesting that inhibiting BPTF could provide a novel immunotherapy.

## INTRODUCTION

To escape antitumor immunity tumor cells suppress natural killer (NK) cell activity [1]. Toward this end, tumor cells alter ligand presentation to NK cell receptors by acquiring genetic and epigenetic changes to the genome [2]. Understanding how epigenetics affects these changes is important because epigenetic modifications are reversible and, therefore, could be corrected with therapeutics [3].

One class of NK cell activating receptors are the natural cytotoxicity receptors (NCRs)[4]. There are 3 NCRs in humans (NKp30, NKp44, and NKp46) and 1 in mice (NCR1). These receptors can control tumor growth *in vivo*, and promote cytolytic activity to cancer cells *in vitro* [5–8]. Both human and mouse NCRs recognize heparan sulfate (HS) chains on cell surface heparan sulfate proteoglycans (HSPG)[9]. These HS chains bind growth factors, cytokines and proteins to regulate a variety of biological processes [10]. In mammals, HS are removed from HSPGs by heparanase to release bound factors and reorganize the extracellular matrix. In most normal cells *HPSE* expression is low, but it is commonly upregulated in many cancers to promote cell growth, motility, metastasis and inflammation [11].

One epigenetic regulator is the ATP-dependent chromatin remodeling complex, nucleosome remodeling factor (NURF). In mammals it is composed of 3 subunits: bromodomain PHD-finger containing transcription factor (BPTF), which is both essential and unique to NURF; the ISWI ATPase SNF2L; and the WD repeat protein pRBAP46/48 [12–14]. NURF slides nucleosomes *in cis* to alter accessibility of DNA for transcription factor binding, which ultimately regulates gene expression [12]. NURF is essential for embryonic development but is not cell essential [15, 16]. The BPTF gene is frequently amplified and overexpressed in a variety of cancers including breast, lung, and brain [17], though how NURF functions in cancer biology is just beginning to be understood. To better understand how epigenetic regulators, and NURF in particular, influence tumor biology, we pursued a loss of function approach using well established syngeneic breast cancer models.

## RESULTS

### NK cell-mediated antitumor immunity is enhanced to BPTF-depleted breast tumors

**Figure 1:**
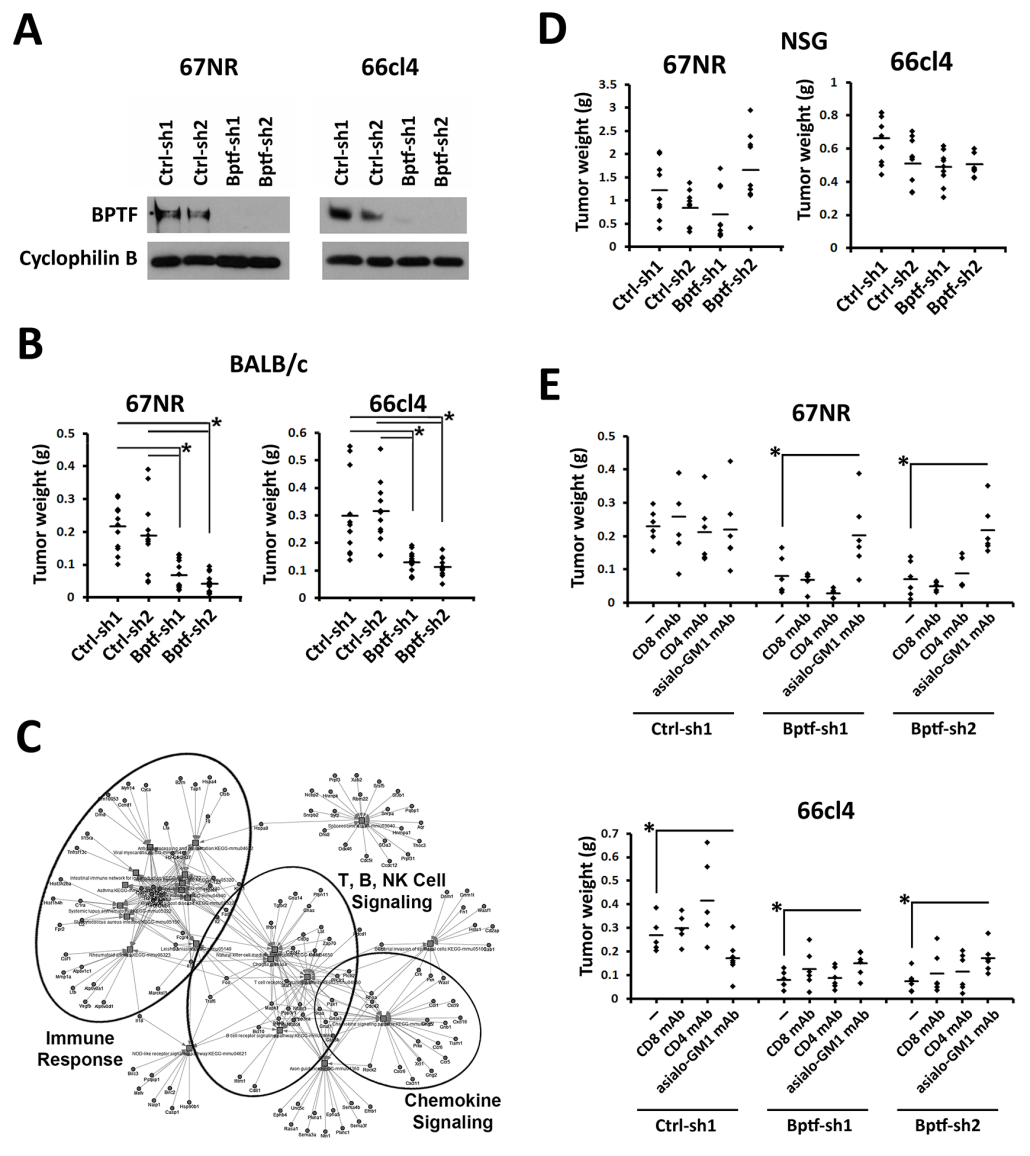
NK cells are required to reduce BPTF KD 67NR and 66cl4 tumor weight **(A)** BPTF Western blot analysis from control (Ctrl-sh1, Ctrl-sh2) and BPTF KD (Bptf-sh1, Bptf-sh2) 67NR and 66cl4 total cell extracts. Cyclophilin B is used as a loading control. **(B)** 67NR and 66cl4 tumor weights harvested from BALB/c mice (n ≥ 11 biological replicates, * = ttest pvalue < 0.003). **(C)** Low resolution KEGG pathway analysis of 67NR and 66cl4 significantly deregulated genes highlighting clusters of genes with function in the immune system (For high resolution please refer to Supplementary Figure 2B). **(D)** 67NR and 66cl4 tumor weights harvested from NSG mice (n = 9 biological replicates). **(E)** 67NR and 66cl4 tumor weights harvested from undepleted, or CD8, CD4 or asialo-GM1 mAb depleted BALB/c mice (n = 6 biological replicates, * = ttest pvalue < 0.05). Some dots overlap.

To investigate roles for NURF in cancer cell biology, we transduced the well-established 67NR and 66cl4 mouse breast cancer cell lines [18] with retroviruses expressing control (Ctrl-sh1 or Ctrl-sh2) or BPTF shRNAs (Bptf-sh1 or Bptf-sh2) (Figure 1A). BPTF knockdown (KD) was used to deplete NURF because it is unique and essential to the complex [13, 14]. In culture we observed equivalent doubling times, cellular morphology, and levels of apoptosis (Supplementary Figure 1A-1C). To discover novel roles for BPTF in tumor biology, we transplanted the 66cl4 or 67NR lines into the 4^th^ mammary fat pad of syngeneic BALB/c mice. After 3-4 weeks, we observed reduced BPTF KD tumor weight (Figure 1B). Tumor weights were used instead of volume to measure growth because BPTF KD tumors grow flat, confounding volume-based comparisons to controls [19]. Microarray expression profiling of control and BPTF KD tumors discovered an enrichment of genes with gene ontology (GO) terms which included immune response descriptors (Supplementary Figure 2A; Supplementary Data Set 1). In agreement with microarray data, KEGG analysis of a combined gene list from both tumor types identified an abundance of genes involved in the immune response (Figure 1C; for high resolution see Supplementary Figure 2B; Supplementary Data Set 1) [20]. To confirm the importance of the immune response for BPTF KD tumor growth, we repeated our tumor studies in an immune-deficient NOD/SCID, Ifrg2r−/− (NSG) background [21]. These experiments showed equivalent BPTF KD tumor weights to controls, demonstrating the immune system is required to reduce the growth of BPTF KD tumors (Figure 1D).

To identify immune cells that are important for reducing BPTF KD tumor growth, we repeated our tumor studies in mice depleted of NK cells, CD8+ T cells, or CD4+ T cells by monoclonal antibody (mAb) treatments. We observed improved growth of 67NR and 66cl4 BPTF KD tumors with NK cell depletion (anti-asialo-GM1 mAb), but not with CD8+ or CD4+ T-cell depletion, indicating that NK cells are required for reduced BPTF KD tumor growth (Figure 1E) (Supplementary Figure 3A-3C).

**Figure 2:**
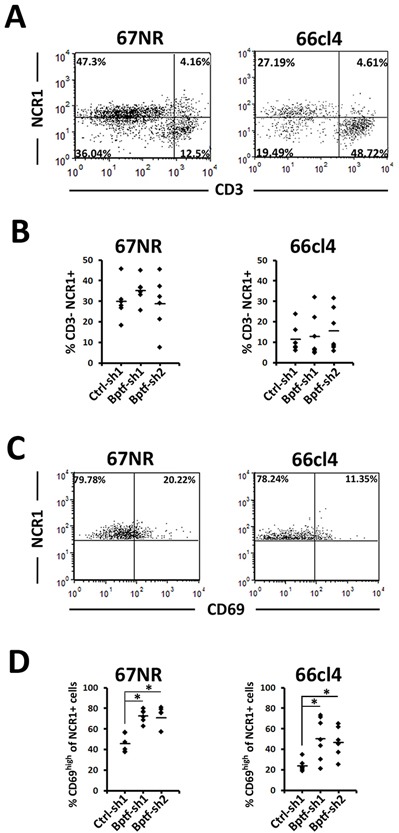
NK cells are more active in BPTF depleted tumor microenvironments **(A)** Representative flow cytometry dot plots of live tumor infiltrating NK cells stained with CD3ε and NCR1. **(B)** Percentages of live CD3ε-, NCR1+ NK cells from A (n ≥ 6 biological replicates). **(C)** Representative flow cytometry dot plots of live tumor infiltrating NK cells stained with CD69 and NCR1. CD69^high^ is defined by CD69 staining of live CD3ε- NCR1+ cells from the spleen. **(D)** Percentages of live NCR1+ cells that are CD69high (n = 6 biological replicates, * = ttest pvalue < 0.006). Quantitative data shown represent mean ± stdev.

We next examined the abundance and activation of NK cells in the BPTF KD tumor microenvironment (TME)[22]. This analysis showed a greater population of NK cells (CD3-, NCR1+) in 67NR tumors compared to 66cl4 tumors (Figure 2A). While present, the CD3-, NCR1+ populations did not change with BPTF KD in 66cl4 or 67NR TME (Figure 2B). However, we observed a greater percentage of NCR1+, CD69^high^ cells in 67NR and 66cl4 BPTF KD tumors, demonstrating that a greater fraction of NK cells in BPTF KD tumors are activated as defined by CD69 expression (Figure 2C and 2D).

### NK cell cytotoxic activity is enhanced to BPTF depleted cancer cells *in vitro*

**Figure 3:**
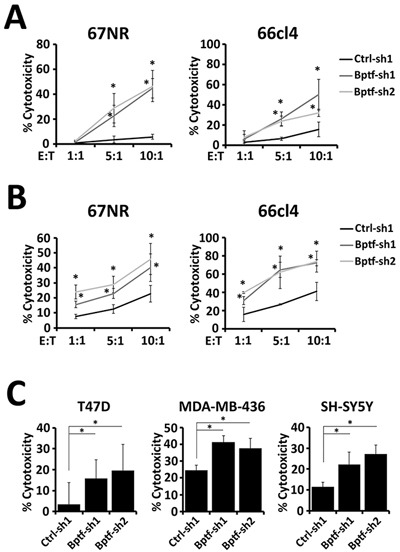
NK cells have greater cytolytic activity to BPTF KD targets **(A-C)** Percent target cell cytolytic activity by LDH assay. **(A)** NK cells from naive BALB/c mice were cocultured with targets at the indicated effector:target (E:T) ratios (n = 3 biological replicates, * = ttest pvalue < 0.05). **(B)** NK-92 cells were cocultured with targets at the indicated E:T ratios (n = 3 biological replicates, * = ttest pvalue < 0.05). **(C)** NK-92 cells were cocultured with human targets at a 10:1 E:T ratio (n ≥ 3, * = ttest pvalue < 0.05). Quantitative data shown represent mean ± stdev.

Coculture of purified naive mouse NK cells with 67NR and 66cl4 cells showed enhanced activation and cytolytic activity to BPTF KD targets (Figure 3A) (Supplementary Figure 4A-4D). To determine if BPTF KD cells are more sensitive to NK cell-mediated killing, we stimulated their cytolytic activities independent of ligand using PMA + ionomycin (P+I). P+I activated NK cells killed control and BPTF KD targets equivalently, indicating that BPTF KD targets are not more sensitive to lysis by NK cells (Supplementary Figure 4E). To determine if soluble factors enhance NK cell activity, we pre-incubated naive mouse NK cells with media from cultured control or BPTF KD 67NR or 66cl4 tumor cells prior to their use in the cytolytic assay. From these experiments, we observed equivalent NK cell cytolytic activity toward BPTF KD targets with media preconditioned with either control or BPTF KD cells (Supplementary Figure 4F). These results in combination demonstrate that changes on the surface of BPTF KD tumor cells, as opposed to a soluble factor, improve NK cell activation and cytolytic activity.

We next determined if the enhanced NK cell cytolytic activity to BPTF KD targets is conserved between mice and humans. Toward this end, we cocultured the human NK-92 cell line [23] with either mouse (67NR, 66cl4) or human (T47D, MDA-MB-436, SH-SY5Y) BPTF KD targets (Supplementary Figure 4G). From these experiments we observed enhanced cytolytic activity to all BPTF KD cell lines, indicating that changes leading to enhanced cytolytic activity are conserved (Figure 3B and 3C).

### BPTF regulates cell surface HSPG abundance and *Hpse* expression

**Figure 4:**
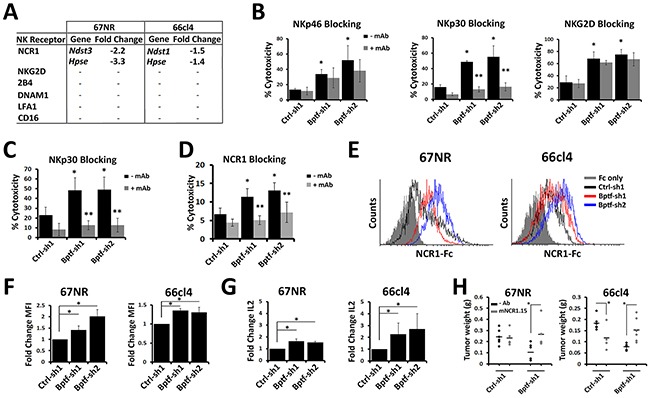
NK cell cytolytic activity to BPTF KD cells requires NCRs **(A)** Changes in NK receptor ligand expression measured by microarray in BPTF KD tumors harvested from NSG mice compared to controls. Hpse was measured independently by qRT-PCR because it was not on the microarray (* = ttest pvalue < 0.00002). **(B-D)** Percent target cell cytolytic activity by LDH assay. (B) NK-92 cells pretreated with anti-NKp46, anti-NKp30 or anti-NKG2D mAb were cocultured on 66cl4 targets at a 10:1 E:T ratio (n = 3 biological replicates, * = ttest pvalue < 0.05). (C) NK-92 cells pretreated with anti-NKp30 were cocultured with T47D targets at a 10:1 E:T ration (n = 3 biological replicates, * = ttest pvalue < 0.05). **(D)** Mouse NK cells pretreated with anti-NCR1 blocking mAb (clone mNCR1.15) were cocultured with 66cl4 targets at a 5:1 E:T ratio (n = 3 biological replicates, * = ttest pvalue < 0.04). (* = significant to no mAb Ctrl-sh1, ** = significant to the respective no mAb hairpin). **(E)** Representative flow cytometry histograms of NCR1-Ig binding to 67NR and 66cl4. **(F)** Fold change of MFI from D (n = 3 biological replicates, * = ttest pvalue < 0.03). **(G)** qRT-PCR analysis of *Il-2* expression in Ncr1-ζ, *Il-2* reporter BW cells incubated with control or BPTF KD cells for 48 hrs (n ≥ 3 biological replicates, * = ttest pvalue < 0.05). **(H)** 67NR and 66cl4 tumor weights harvested from untreated or mNCR1.15 treated BALB/c mice (n ≥ 4 biological replicates, * = ttest pvalue < 0.05). All quantitative data shown represent mean ± stdev.

We conducted microarray gene expression ana-lyses of control and BPTF KD 67NR and 66cl4 tumors from NSG mice to identify candidate BPTF-regulated NK cell receptor ligands. Analysis of these data sets focused on activating receptor ligands because they are conserved between mice and humans [24]. This is not true of the inhibitory MHC ligands which are species restricted in activity [25], and not significantly BPTF regulated in 67NR or 66cl4 cells (Supplementary Figure 5A). Combined qRT-PCR and microarray experiments identified three BPTF regulated genes with functions in HS metabolism (*Ndst1, Ndst3, Hpse*) but did not identify any known activating NK cell receptor ligands (Figure 4A; Supplementary Figure 5B; Supplementary Data Set 1). HS and HSPGs are well characterized co-ligands for the NCRs, which are required for efficient NK cell-mediated tumor cell killing [26, 27].

Because HS is a known co-ligand to all NCRs, it is plausible that the enhanced NK cell cytolytic activity to BPTF KD cells occurs through the NCRs. mAb blocking of NKp30, but not NKp46 or NKG2D, on NK-92 cells inhibited the enhanced cytolytic activity against BPTF KD 66cl4 targets (Figure 4B). NKp44 was not included in our blocking studies because NK-92 cells do not express NKp44 [28]. NKp30 is highly expressed and is a major NCR used by NK-92 cells for antitumor activity [28, 29]. Functions for NKp30 in promoting NK cell activity to BPTF KD cells is conserved as demonstrated by reduced NK-92 cytolytic activity toward BPTF KD T47D targets with NKp30 blocking (Figure 4C). Similar reductions in mouse NK cell cytolytic activity was observed after NCR1 mAb blocking, the only NCR expressed in mouse NK cells (Figure 4D) [30]. Also, the addition of the NCR competitive inhibitor heparin [9] erased the NK-92 and mouse NK cell cytolytic activity against 67NR and 66cl4 BPTF KD targets (Supplementary Figure 5C and 5D).

We next used a recombinant NCR1-Ig fusion protein to measure the abundance of NCR1 ligands on the surface of BPTF KD 67NR and 66cl4 cells. From these experiments, we discovered enhanced binding of NCR1-Ig to BPTF KD cells (Figure 4E and 4F). We then measured NCR1 activation using a cell line with an *Il-2* reporter regulated by an NCR1-ζ fusion receptor [31]. Coculture experiments show elevated *Il-2* gene expression when in contact with BPTF KD 67NR and 66cl4 cells (Figure 4G) (Supplementary Figure 5E), further suggesting that enhanced NK cell cytolytic activity results from increased NCR1 activity. We next used the NCR1 blocking mAb in tumor bearing mice and observed that BPTF KD tumor weights are rescued with NCR1 blocking (Figure 4H). As previously reported after NCR1 blocking mAb treatments NK cells are not depleted and cell surface NCR1 decreases (Supplementary Figure 5F and 5G)[32].

**Figure 5:**
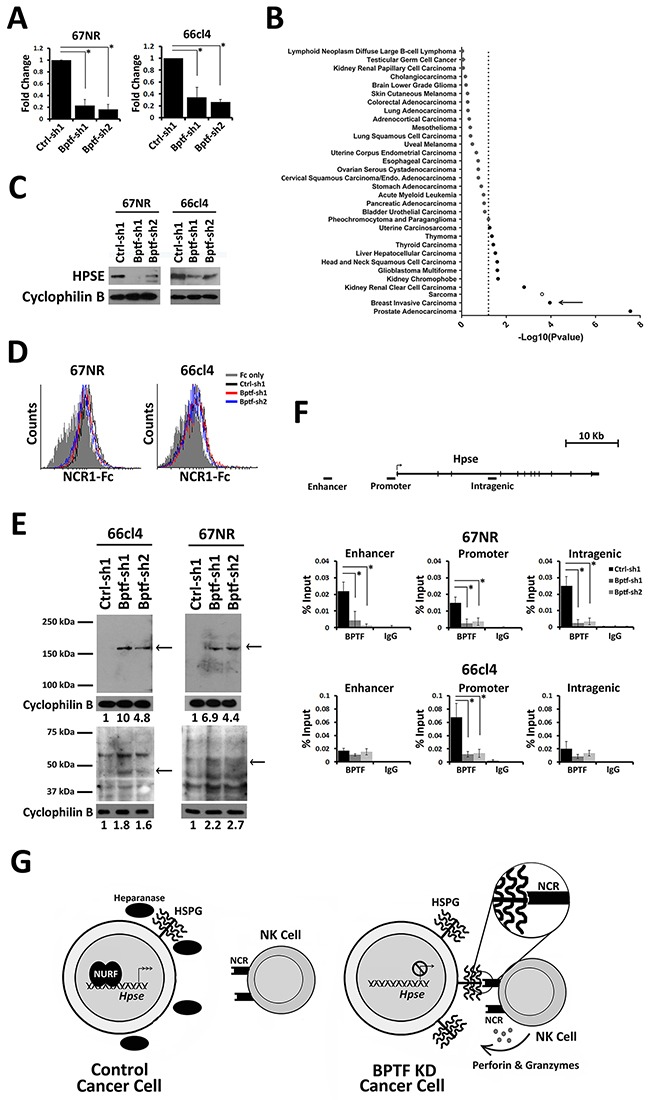
BPTF regulates *Hpse* expression **(A)** qRT-PCR analysis of *Hpse* expression from 67NR and 66cl4 cells (n = 3 biological replicates, * = ttest pvalue < 0.006). **(B)** Differential *Hpse* expression in BPTF high and BPTF low expression groups from TCGA datasets. The negative base 10 log of the p-value for each t-test is shown. Dashed line: significance cutoff pvalue = 0.05. Filled circles: positive correlation. Outlined circles: negative correlation. Breast cancer data set is shown with arrow. **(C)** Western blot analysis of cell surface HPSE from control and BPTF KD 67NR and 66cl4 cells. Cyclophilin B was used as a normalization control. **(D)** Representative flow cytometry histogram of NCR1-Ig binding to bacterial heparinase treated tumor cells. **(E)** Western blot analysis of cell surface HSPG using anti-HS primary mAb. Cyclophilin B was used as a normalization control. Arrows: reproducible changes in HSPG abundance with BPTF KD. ImageJ relative quantitation to controls are shown as numbers below blots. **(F)** BPTF ChIP at mouse *Hpse* in control and BPTF KD 67NR and 66cl4 cell lines (n = 3 biological replicates, * = ttest pvalue < 0.04). **(G)** A Model: In tumor cells NURF stimulates *Hpse* expression, and as a result cell surface heparanase abundance. Increased heparanase abundance reduces cell surface HSPGs and NCR HS co-ligand abundance, inhibiting NK-cell antitumor activity. When NURF is depleted, *Hpse* expression and heparanase is reduced. Reduced heparanase abundance increases cell surface HSPGs and NCR HS co-ligands, improving NK cell-mediated antitumor activity. All quantitative data shown represent mean ± stdev.

Our expression analysis identified *Hpse*, the major regulator of cell surface HS abundance, as a BPTF-regulated gene in both 67NR and 66cl4 BPTF KD tumors (Figure 4A)[33]. These results were confirmed in culture for the 67NR, 66cl4, T47D, SH-SY5Y, and MDA-MB-436 cell lines (Figure 5A) (Supplementary Figure 5H). In addition, an analysis of *BPTF* and *HPSE* expression in human cancers from the TCGA data sets revealed a highly significant correlation between high *BPTF* expression and high *HPSE* expression in several cancer types including breast (see arrow) (Figure 5B). Consistent with *Hpse* gene expression, Western blotting discovered significant reductions in cell surface heparanase abundance with BPTF KD (Figure 5C). The importance of heparanase for enhanced NK cell activity to BPTF KD cells was investigated using bacterial heparinase treatments and heparanase KD cell lines. Pretreatment of tumor cells with bacterial heparinase suppressed the enhanced binding of NCR1-Ig to BPTF KD cells (Figure 5D) and reduced the enhanced cytolytic activity of NK cells to 66cl4 BPTF KD targets (Supplementary Figure 5I). Similarly, we observed that heparanase KD in control 66cl4 cells results in enhanced NK cell cytolytic activity, consistent with heparanase abundance regulating NK cell activity (Supplementary Figure 5J and 5K). To determine if decreased *Hpse* expression with BPTF KD correlates with changes in cell surface HSPGs, we measured HSPG abundance by Western blotting cell surface protein extractions using an antibody to HS. Three HSPGs from these experiments had reproducibly increased levels in BPTF KD cells: a ∼150 kDa band, a ∼55 kDa band and a ∼45 kDa band (Figure 5E, see arrows). It is unlikely that expression of the HSPG core proteins are BPTF-dependent because they were not BPTF-dependent from our microarray analyses (Supplementary Figure 5L). Additionally, only the MMP2 HSPG sheddase was deregulated in 67NR, but not 66cl4, BPTF KD NSG tumors, suggesting that HSPG cell surface shedding is not BPTF-dependent (Supplementary Figure 5L). In addition, NURF occupies regulatory elements of *Hpse* because chromatin immunoprecipitation (ChIP) revealed BPTF occupancy broadly localized at the *Hpse* gene in 67NR and localized at the *Hpse* promoter in 66cl4 cells (Figure 5F) [34]. From these results, we propose a model where NURF activates *Hpse* in cancer cells, either directly or indirectly, elevating cell surface heparanase and reducing cell surface HSPGs. Because HSPGs are known co-ligands for NCRs, our model predicts that NURF suppresses NCR-mediated NK cytolytic activity by upregulating heparanase levels (Figure 5G).

### BPTF depletion enhances the immune response to established tumors

**Figure 6:**
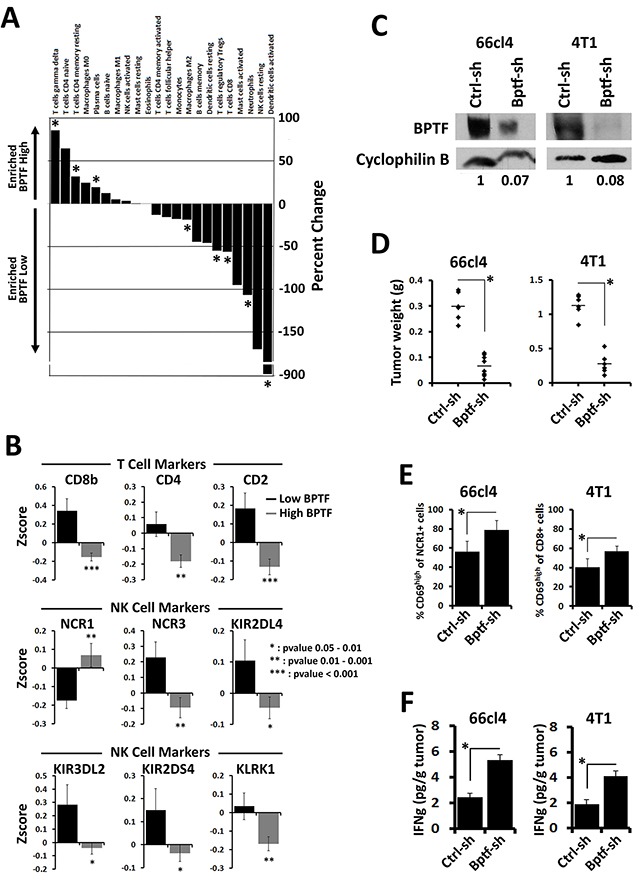
BPTF is required for an immune suppressive tumor microenvironment **(A)** Differential immune profiles identified using CIBERSORT between *BPTF* high and *BPTF* low expression groups from microarray expression profiles (* = ttest pvalue < 0.1). **(B)** Differential expression of immune cell markers in *BPTF* high and *BPTF* low expression groups from Zscore normalized expression data from breast cancer TCGA datasets (pvalues are shown in panel). **(C)** BPTF Western blot analysis of 66cl4 and 4T1 tumors injected with rAd expressing either control or BPTF KD shRNAs. Cyclophilin B was used as a loading control. ImageJ relative quantitation to controls are shown as numbers below blots. **(D)** Weights of 66cl4 and 4T1 tumors after treatment with rAd (n ≥ 6 biological replicates, * = ttest pvalue < 0.000003). **(E)** Flow cytometry analysis of 66cl4 tumor infiltrating NK cells and 4T1 tumor infiltrating CD8+ T cells. Active lymphocytes are quantified as percent CD69high of total infiltrating NK cells (NCR1+) or CD8+ T cells (CD8+) (n ≥ 4 biological replicates, * = ttest pvalue < 0.01). **(F)** IFNγ concentrations from 66cl4 and 4T1 tumor extracts measured by ELISA (n ≥ 6 biological replicates, * = ttest pvalue < 0.04). All quantitative data shown represent mean ± stdev.

To determine if BPTF suppression of the antitumor immune response could be conserved in human tumors, we monitored the immune cell composition from human breast tumor microarray data sets using the CIBERSORT algorithm [35]. From this analysis, we observed an enrichment of γδ T cells, resting CD4 T cells and B cells in tumors with high *BPTF* expression. Conversely, tumors expressing low levels of *BPTF* are enriched for active dendritic cells, macrophages, neutrophils, CD8+ T cells and Treg cells (Figure 6A). These results suggest that low *BPTF* levels correlate with increased abundance and activity of immune cells in human breast tumors. These results are supported by an independent analysis of the larger breast cancer provisional TCGA RNA-Seq data set [36]. This analysis revealed an increase in the expression of CD8 T cell, NK cell, macrophage and Treg cell markers, and immune cell cytokines in breast tumors with low levels of BPTF expression (Figure 6B) (Supplementary Figure 6A). We observe increased *NKp44* (*NCR3*) expression but not *NKp46* (*NCR1*) possibly because the elevated *IL2* expression in *BPTF* low tumors could downregulate *NKp46* [37].

To further test this model, we injected established 66cl4 and 4T1 tumors with replication-deficient adenovirus (rAd) expressing control or BPTF KD shRNA. 67NR tumors could not be used for these experiments because they undergo apoptosis after transduction by rAd in culture (data not shown). We had previously shown that BPTF KD 4T1 tumors upregulate antigen processing genes to improve the CD8+ T cell antitumor response [19]. Western blot analysis of 66cl4 and 4T1 treated tumors show that rAd treatments resulted in BPTF KD (Figure 6C). BPTF KD in established tumors significantly reduced tumor weights compared to controls (Figure 6D), and we occasionally observed complete 66cl4 tumor regression with BPTF KD (rAd BPTF shRNA = 3 of 17 treated tumors, rAd control shRNA = 0 of 16 treated tumors). To measure the effects of BPTF KD on the antitumor immune response, we monitored the activation of NK and CD8+ T cells in 66cl4 and 4T1 tumors, respectively. Consistent with our previous studies, BPTF KD in established tumors resulted in a greater percentage of CD69^high^ NCR1+ NK cells in the 66cl4 TME, or CD69^high^ CD8+ T cells in the 4T1 TME (Figure 6E). Increased activation of NK and CD8+ T cells coincided with elevated IFNγ, suggesting a conversion to a Th1 TME (Figure 6F). As a control, transduction of NK-92 cells and splenic mouse OT1 T cells in culture with rAd to achieve BPTF KD followed by coculture assays show that BPTF KD does not significantly alter NK or T cell cytolytic activity (Supplementary Figure 6B-6F). These results indicate that these effector cells are likely functional after BPTF KD rAd treatments.

## DISCUSSION

Understanding how tumors escape the immune response is of great interest to tumor immunologists. Epigenetic escape mechanisms, most prominently DNA methylation and histone deacetylation, suppress tumor cell antigenicity to inhibit T cells, and ligand expression to inhibit NK cells [38]. Because many epigenetic regulators are required for gene expression, chromatin remodeling is predicted to have similar functions in tumor cells. Using transplantable tumor models, we tested this prediction and discovered that BPTF, and by extension NURF, represses NK-mediated antitumor activity. We also observed pro-tumor NK cell activities to 66cl4 tumors, which the use of a NCR1 blocking mAb suggest are NCR1 mediated. NCR1 expressing NK cells and immature lymphoid cells can control the immune response by direct killing of dendritic cells and T cells [39, 40], which could suppress an adaptive immune response to 66cl4 tumors, possibly explaining their pro-tumor activities. In total, these results support the idea that BPTF is a novel regulator of the NK cell-mediated antitumor immune response.

To identify BPTF-dependent NK receptor ligands, we used two complementary approaches. We first used the human NK-92 cell line to demonstrate that the BPTF-regulated ligand(s) are conserved across species, and second used microarrays to discover that components of the HS synthesis and degradation pathways are BPTF-dependent in tumors. The most noteworthy BPTF-regulated factor discovered was the HS degrading enzyme heparanase, an enzyme which is highly conserved in both sequence and function between mouse and humans [41]. NURF could directly regulate *Hpse* expression because BPTF occupies its regulatory elements, but it is also equally possible that NURF regulates *Hpse* indirectly. Characterizing NURF-regulated chromatin remodeling activities and mechanisms of NURF recruitment to these regulatory elements would be required to support a model where NURF directly regulates *Hpse*. Using similar approaches we discovered that NURF directly regulates chromatin structures surrounding CTCF binding sites to influence their regulatory activity [15]. One possibility is that NURF could be recruited to the *Hpse* promoter through its ability to bind the promoter associated histone modifications H3K4me3 and H4K16ac [42]. Alternatively, NURF could be recruited to *Hpse* regulatory elements through interactions with novel transcription factors [12].

HS and HSPGs are highly conserved, common co-ligands to all NCRs, and they influence NK cell-mediated antitumor immunity in both humans and mice [5, 9, 11]. Consistent with BPTF regulating the expression of NCR co-ligands, NKp30 and NCR1 mAb blocking and heparin inhibition experiments reduce the enhanced NK cell cytolytic activity to BPTF KD targets *in vitro*. Also consistent, we observe rescue of BPTF KD tumor growth when NCR1 blocking mAb are used *in vivo* and is consistent with other reports that NCR1 is important for antitumor activity [5, 43, 44]. Coincident with reduced cell surface heparanase, Western blotting identified HSPGs which increase in abundance on the surface of BPTF KD cells. Heparanase binds HSPGs, stimulating endocytosis and eventually HS cleavage, which could explain the observed increased cell surface HSPG levels on BPTF KD cells [41]. These results in total suggest a model where BPTF upregulates heparanase expression to down regulate cell surface HSPG levels, a co-ligand to NCR1, reducing NK cell cytolytic activity. While this model is consistent with the data we cannot rule out that bonified, and yet unidentified, NCR ligands are NURF regulated which contribute to changes in NK cell activity [45].

The significance of BPTF, and by extension NURF, regulation of *HPSE* in cancer could have broader implications. *HPSE* is commonly upregulated in cancer cells to impact angiogenesis, metastasis, tumor growth and inflammation [11]. It is therefore plausible that differences in tumor shape with BPTF KD could result from abnormal angiogenesis, or establishing a fibrin shell. Decreases in heparanase levels could also contribute to defects in metastases, as previously observed with BPTF KD melanoma tumors [46]. Defects could result from reductions in cell motility [45], or from enhancements of NK cell antitumor activity which is most significant to metastatic cancer cells [47]. These hypotheses, partially supported by our data, warrant more detailed studies on roles for BPTF during metastasis.

NK cells have greatest therapeutic significance to hematological malignancies, where NK cell activity can result in disease remission. NK cell activity against solid tumors is less significant because the immune suppressive TME inhibits NK cell infiltration and activity [48]. We show for the first time that depleting BPTF in the TME could circumvent problems inherent to the use of NK cells as an immunotherapy toward solid tumors. BPTF depletion in 66cl4 tumors with rAd resulted in tumor regression with about 18% frequency, despite incomplete BPTF KD. Regression could result from the elevated IFNγ converting the TME to a Th1 microenvironment and enhancing the activity of other effector cells, or from promoting epitope spreading, which could expand novel tumor reactive T cell clones [49]. We speculate that tumor regression is not observed in the 4T1 model because of significant immunosuppressive activity of the remaining myeloid derived suppressor cells after gemcitabine treatments [50]. It is also noted that there are observed differences in the immune response between mouse and human BPTF KD vs *BPTF* low tumors. Using the mouse tumor models we do not detect significant differences in NK cell abundance with BPTF KD, but differences in abundance are observed in human breast tumors with low *BPTF* expression. These differences could be due to differences in how mouse tumors or NK cells respond to low intratumor BPTF levels compared to similar conditions in humans. Alternatively, increased infiltration of NK cells in *BPTF* low human breast tumors could result from increased CD8 T cell antitumor activity, which we previously observed to the 4T1 mouse breast tumor model [19]. To resolve these unknowns, expanded studies on the response of human tumors to BPTF inhibition will need to be conducted, which was limited in the current study to the use of cancer cell lines *in vitro*. It is our ultimate goal to translate these findings into a novel immunotherapy, which could be initiated by using newly identified BPTF bromodomain small molecule inhibitors [51] alone or in combination with established immune stimulating chemotherapies [52] or immunotherapies like NK cell adoptive cell transfer [53].

## MATERIALS AND METHODS

### Mice

BALB/cJ, NOD/SCID/Ifrg2r−/− (NSG) and C57BL/6-Tg(TcraTcrb)1100Mjb/J (OT1) female mice 6-8 weeks of age weighting ∼ 20 g (Jackson Laboratory) were housed under aseptic barrier conditions as approved by Virginia Commonwealth University IACUC.

### Cell culture

67NR, 66cl4, 4T1 (Wayne State University, 2010) SY5Y and HEK 293T (ATCC, 2010) were cultured in complete media (CM) (DMEM, 10% FBS, 1% NEAA, 1% glutamine, and 1% penicillin/streptomycin). T47D and MDA-MB-436 (ATCC, 2010) were cultured in RPMI, 10% FBS, 1% NEAA, 1% glutamine, 1% penicillin/streptomycin and 5 μg/ml insulin. NK-92 cells (ATCC, 2012) were cultured in Alpha MEM, 12.5% horse serum, 12.5% FBS, 2 mM L-glutamine, 1.5 g/L sodium bicarbonate, 0.2 mM inositol, 0.1 mM 2-mercaptoethanol, 0.02 mM folic acid and 200 U/ml Il-2. OT1 T cells were cultured in CM with 1% HEPES, 5×10^−5^ M 2-mercaptoethanol and 500 U/ml mouse Il-2. Cell lines were validated by Wayne State University or ATCC prior to shipment by short tandem repeat (STR) profiling. Mycoplasma contamination was tested and confirmed to be negative every 2 years using Universal Mycoplasma Detection Kit (ATCC).

ShRNAs were introduced into 67NR and 66cl4 cells using the pSIREN-RetroQ system (Clonetech). pSIREN plasmids Ctrl-sh1, Ctrl-sh2, Bptf-sh1, Bptf-sh2, HPSE-sh1, HPSE-sh2 are available at Addgene as stock numbers 73665, 73666, 73667, 83045, 92033, 92034 respectively. ShRNAs were introduced into T47D, MDA-MB-436 and SH-SY5Y cells using the lentiviral pLVTHM system. pLVTHM plasmids Ctrl-sh1, Bptf-sh1, Bptf-sh2 are available at Addgene as stock numbers 83046, 83277, 83278, respectively. Transduced cells were selected with 0.5 μg/ml puromycin after 48 hours.

### Replication-deficient adenovirus

To construct rAd-BPTF and rAd-Luc, we used the AdenoQuick cloning system (OD260, Inc.) according to the manufacturer's instructions. pAD388 cosmids Ctrl-sh, Bptf-sh are available at Addgene as stock numbers 83275, 83276, respectively. The cosmid DNA was transfected into HEK-293T cells and serially amplified 3 times to obtain high titer virus [54]. Virus titer was determined by TCID50 according to the manufacturer's instructions. 66cl4 and 4T1 cells were infected with 1×10^6^ PFU rAd twice during a one week period.

Spleens from OT1 mice were harvested and depleted of erythrocytes with RBC lysis buffer and cocultured with mitomycin C-treated B16F10-OVA cells for 3 weeks. 3×10^6^ cultured NK-92 or 5×10^5^ expanded OT1 T cells were then infected with 2×10^7^ or 2×10^6^ rAd in a volume of 10 ml or 1 ml for 5 days.

### Tumor studies

1×10^5^ 67NR cells and 1×10^4^ 66cl4 cells were injected into the fourth mammary fat pad of BALB/c or NSG mice. Tumors were analyzed at 21 days (67NR) or 28 days (66cl4). For rAd studies, BALB/c mice were inoculated with 1×10^6^ 66cl4 or 3×10^5^4T1 cells and injected intratumorally with rAd-Luc or rAd-BPTF (1×10^9^ PFU in 100ul) every 3 days for 3 weeks once tumors were ∼5 mm in any dimension. Animals were euthanized 1 week after the last rAd injection. For 4T1 studies, mice were injected intraperitoneally (I.P.) with 1.2 mg/mouse gemcitabine 5 days after tumor inoculation and once every 7 days thereafter.

### mAb depletions

GK1.5, 2.43 and SH-34 mAb depletions were performed as described previously [19]. Briefly, mAbs were purified from ascites fluid by ammonium sulfate fractionation and 225 μg/mouse was injected on day −2, and −1. Tumors were inoculated on day 0 and mAb was injected once every 5 days following tumor inoculation.

### mNCR1.15 blockade

100 μg/mouse mNCR1.15 (Center for Proteomics, University of Rijeka, Croatia) was injected on day −2 and −1. Cell lines were inoculated on day 0 and mNCR1.15 was injected once every 5 days thereafter.

### Western blot

For total extracts, protein from cell cultures or homogenized tumors was extracted by TRI Reagent. Cell surface protein extraction was performed as described previously [55]. Primary antibodies: anti-BPTF (Millipore Cat# ABE024), anti-HS (Millipore Cat# MAB2040), and anti-HPSE (Santa Cruz Cat# sc25826) then anti-mouse or rabbit HRP secondary (Cell Signaling). Loading was determined Cyclophilin B.

### NK cell cytotoxicity assay

NK cells were purified from the spleen of naïve BALB/c mice by negative selection using MACS separation (Miltenyi Biotech). Purified mouse NK cells or cultured NK-92 cells were placed on mitomycin C-treated targets for 24 hrs (mouse NK cells with mouse targets), 7 hrs (NK-92 cells with mouse targets) or 4 hrs (NK-92 cells on human targets). For antibody blocking experiments, NK cells were pre-incubated for 1 hr with 10 μg/ml blocking antibodies to NCR1 (mNCR1.15 from Stipan Jonjic, Center for Proteomics, University of Rijeka, Croatia), hNKp46 (Biolegend Cat# 13614), hNKp30 (Biolegend Cat# 325204) or hNKG2D (BD Pharmingen Cat# 552866). For hyperactivation, cocultured mouse NK cells were treated with 0.8 μM PMA, 0.35 μM Ionomycin and 50 U/ml IL-2. For media preconditioning, mitomycin C treated targets were incubated for 24 hrs, growth media was then removed and incubated with NK cells for 24 hrs before assay on targets. For heparin competition experiments, cocultures were incubated with 100 μg/ml heparin (Sigma) in growth media during coculture with target cells. For heparinase treatment, 66cl4 cells were pretreated with 1 U/ml bacterial heparinase I/III (Sigma) in DMEM, 1% BSA for one hr at 37°C before coculture with NK cells. Cell death was measured using the CytoTox 96® Non-Radioactive Cytotoxicity Assay (Promega).

### qRT-PCR

Quantitative RT-PCR was performed as described previously [19]. Primer pairs are found in Supplementary Data Set 2.

### Flow cytometry

Cultured 67NR and 66cl4 cells were stained with H2-Kd (Cat# 553566), H2-Dd (Cat# 558917), H2-Ad (Cat# 553548), Qa1 (Cat# 559829) antibodies or Annexin V (Cat# 556421), and 7AAD viability dye. To measure NK activity, mouse NK cells were incubated for 24 hours with mitomycin C treated targets, removed and stained with NCR1 (Cat# 137605) (Biolegend) and CD69 (Cat# 561932) antibodies and 7AAD viability dye. To measure NK cells in the spleen, splenocytes were stained with CD3 and DX5 (Cat#561066) antibodies and 7AAD viability dye.

Tumor infiltrating lymphocytes were purified as described previously [19]. Purified lymphocytes were stained with NCR1, CD69, CD3 (Cat# 553060), or CD8 (Cat# 100706) antibodies and 7AAD viability dye. All antibodies used for flow cytometry are from BD Pharmingen, unless otherwise specified.

The *NCR1*-Ig fusion protein (gift from Ofer Mandelboim from The Hebrew University of Jerusalem) was purified from HEK293T cells as described previously [56]. For heparinase treatment, 67NR and 66cl4 cells were treated with 10 U/ml bacterial heparinase I/III (Sigma) in 1% BSA DMEM for 1 hr at 37°C. Then 1×10^5^ cells were incubated with 1 μg NCR1-Ig for 1 hr at 4°C and stained with PE- anti-human-IgG Fc (Biolegend Cat# 409304).

### Chromatin immunoprecipitation (ChIP)

ChIP was performed as previously described [15]. Primer pairs are found in Supplementary Data Set 2.

### Enzyme linked immunosorbent assay (ELISA)

Tumors were minced and suspended in hanks balanced salts supplemented with complete protease inhibitor. After 3 cycles of freeze thaw, samples were centrifuged at 15,000 x rcf at 4°C for 15 min and the supernatant was collected. ELISA for IFNγ (R&D Systems) was performed according to the manufacturer's instructions.

### NCR1 reporter assay

NCR1-ζ, *Il-2* reporter BW cells (gift from Ofer Mandelboim from The Hebrew University of Jerusalem) were cocultured with 67NR or 66cl4 targets at 1:1 E:T ratio for 48 hrs. *Il2* expression was measured by qRT-PCR and normalized to *NCR1-ζ* expression. Primer pairs are found in Supplementary Data Set 2.

### Microarray

RNA extraction, microarray analysis and statistical analysis were performed as described [57, 58]. Data is available as GEO accession #GSE785756.

### Bioinformatics

RNAseq Zscores were downloaded from The Cancer Genome Atlas (TCGA). For each cancer, patients were divided into high (n = 325) and low (n = 169) *BPTF* expressing groups using a Zscore cutoff of >1 and <-1 respectively. A t-test was performed to evaluate differential expression of HPSE between the *BPTF* high and *BPTF* low groups. For immune cell markers, an unpaired t-test was performed to assess differential expression of immune cell markers between *BPTF* low and *BPTF* high samples.

Microarray expression profiles from patients with breast cancer were downloaded from NCBI GEO (GSE2109) (n = 348). Patients expressing 1 standard deviation above or below the mean level of expression were binned as *BPTF* high (n = 40) and *BPTF* low (n = 45), respectively. Probe level data from both groups were used as input for the CIBERSORT deconvolution algorithm. Immune profiles from *BPTF* high and *BPTF* low groups were compared.

### Statistics

Excel was used to calculate statistical differences between groups using the two-tailed Student's t-test.

## SUPPLEMENTARY MATERIALS FIGURES AND DATA







## Abbreviations

BPTF: bromodomain PHD finger transcription factor
ChIP: chromatin immunoprecipitation
GO: gene ontology
HPSE: heparanase
HS: heparan sulfate
HSPG: heparan sulfate proteoglycan
mAb: monoclonal antibody
NCR: natural cytotoxicity receptor
NK: natural killer
NURF: nucleosome remodeling factor
P+I: PMA + Ionomycin
rAd: replication-deficient adenovirus
TME: tumor microenvironment.
