# Supplementary material for: BPTF inhibits NK cell activity and the abundance of natural cytotoxicity receptor co-ligands
Source: Oncotarget. 2017 May 12;8(38):64344–57. doi: 10.18632/oncotarget.17834 (PMC5610007; doi:10.18632/oncotarget.17834)
Supplement: Supplementary file 1 [file oncotarget-08-64344-s001.pdf]

BPTF inhibits NK cell activity and the abundance of natural cytotoxicity receptor co-ligands

SUPPLEMENTARY MATERIALS

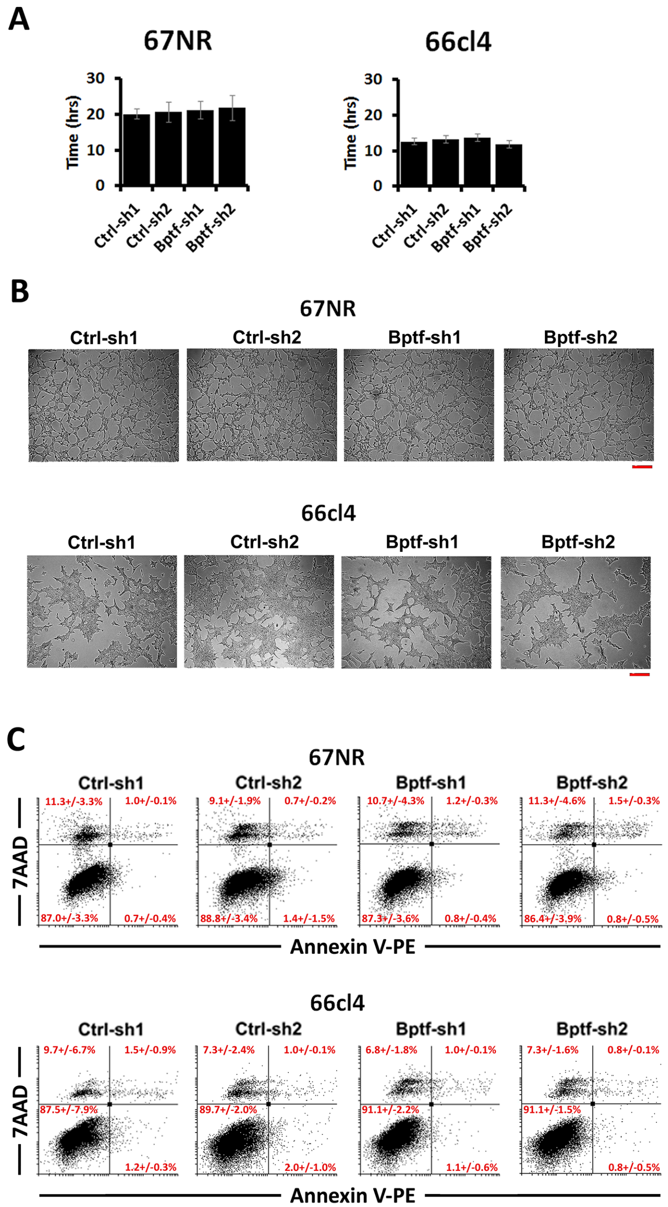

Supplementary Figure 1: BPTF does not affect 66cl4 or 67NR proliferation, cellular morphology or apoptosis *in vitro*. (A) Doubling time of control and BPTF KD 67NR and 66cl4 cultures (n = 3 biological replicates). **(B)** Images of control and BPTF KD 67NR and 66cl4 cells in culture. Scale bar = 150 µm **(C)** Apoptosis was measured on cultured cells using Annexin-V and 7AAD and analyzed by flow cytometry. Populations from each quadrant are shown as percentages with standard deviations for 3 independent measurements. All quantitative data shown represents mean ± stdev.

A

67NR BALB/c

	Gene Count	Benjamini
**GO Term: Biological Process**		
Antigen processing and presentation	13	3.0×10^{-5}
Antigen processing and presentation of peptide antigen	8	1.0×10^{-3}
Immune response	24	2.1×10^{-3}
Antigen processing and presentation of peptide or polysaccharide antigen via MHC class II	6	4.1×10^{-3}
**GO Term: Cellular Component**		
MHC protein complex	10	2.8×10^{-5}
MHC class II protein complex	6	2.9×10^{-5}
TAP complex	4	1.1×10^{-2}
MHC class I peptide loading complex	4	2.0×10^{-2}
Nuclear envelope	10	3.8×10^{-2}
Intracellular organelle lumen	33	4.1×10^{-2}
Organelle lumen	33	3.6×10^{-2}
Endoplasmic reticulum part	12	3.7×10^{-2}
Nuclear lumen	27	4.7×10^{-2}
Membrane-enclosed lumen	33	4.4×10^{-2}

66cl4 BALB/c

	Gene Count	Benjamini
**GO Term: Biological Process**		
Immune response	45	4.9×10^{-2}
**GO Term: Cellular Component**		
Organelle membrane	67	4.3×10^{-2}
Golgi membrane	19	4.3×10^{-2}
Nucleoplasm	52	3.3×10^{-2}
Microtubule	27	2.5×10^{-2}
Golgi apparatus	57	2.5×10^{-2}
Endomembrane system	47	2.6×10^{-2}
Nuclear lumen	69	2.6×10^{-2}
Spliceosome	17	2.9×10^{-2}
**GO Term: Molecular Function**		
Nucleotide binding	153	6.6×10^{-3}
Ribonucleotide binding	126	2.6×10^{-2}
Purine ribonucleotide binding	126	2.6×10^{-2}
GTPase activity	19	1.8×10^{-2}
Purine nucleotide binding	130	1.5×10^{-2}
Tubulin binding	14	1.5×10^{-2}
Cytoskeletal protein binding	40	1.5×10^{-2}
Chromatin binding	20	3.1×10^{-2}
Transcription regulator activity	88	3.1×10^{-2}
GTP binding	34	4.0×10^{-2}

B

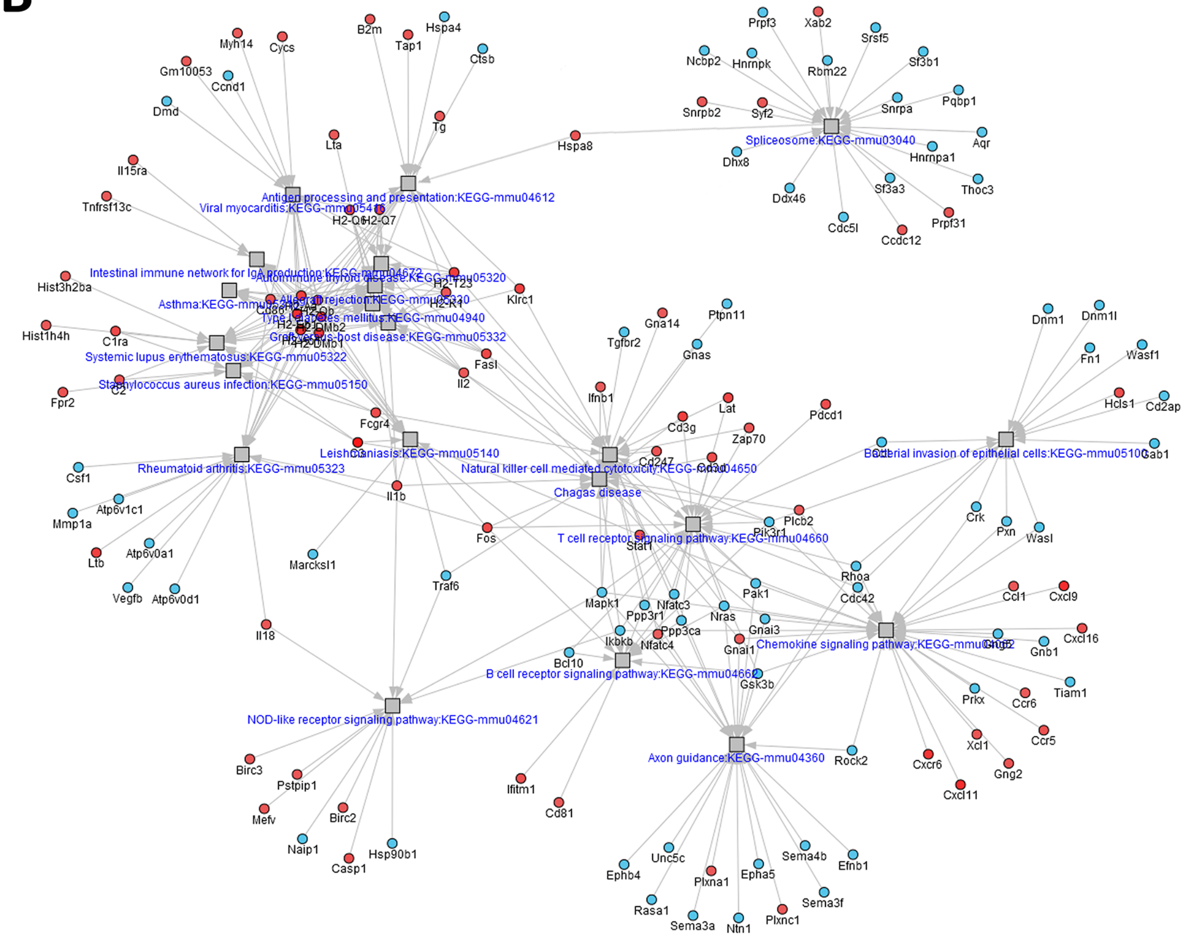

Supplementary Figure 2: BPTF depleted tumors have an enhanced antitumor immune profile. (A) A DAVID gene ontology (GO) analysis on BPTF-dependent genes identified from microarray gene profiling analysis on 67NR and 66cl4 tumors harvested from BALB/cJ mice [59, 60]. **(B)** KEGG pathway analysis of genes identified from A. Red = upregulated, blue = downregulated.

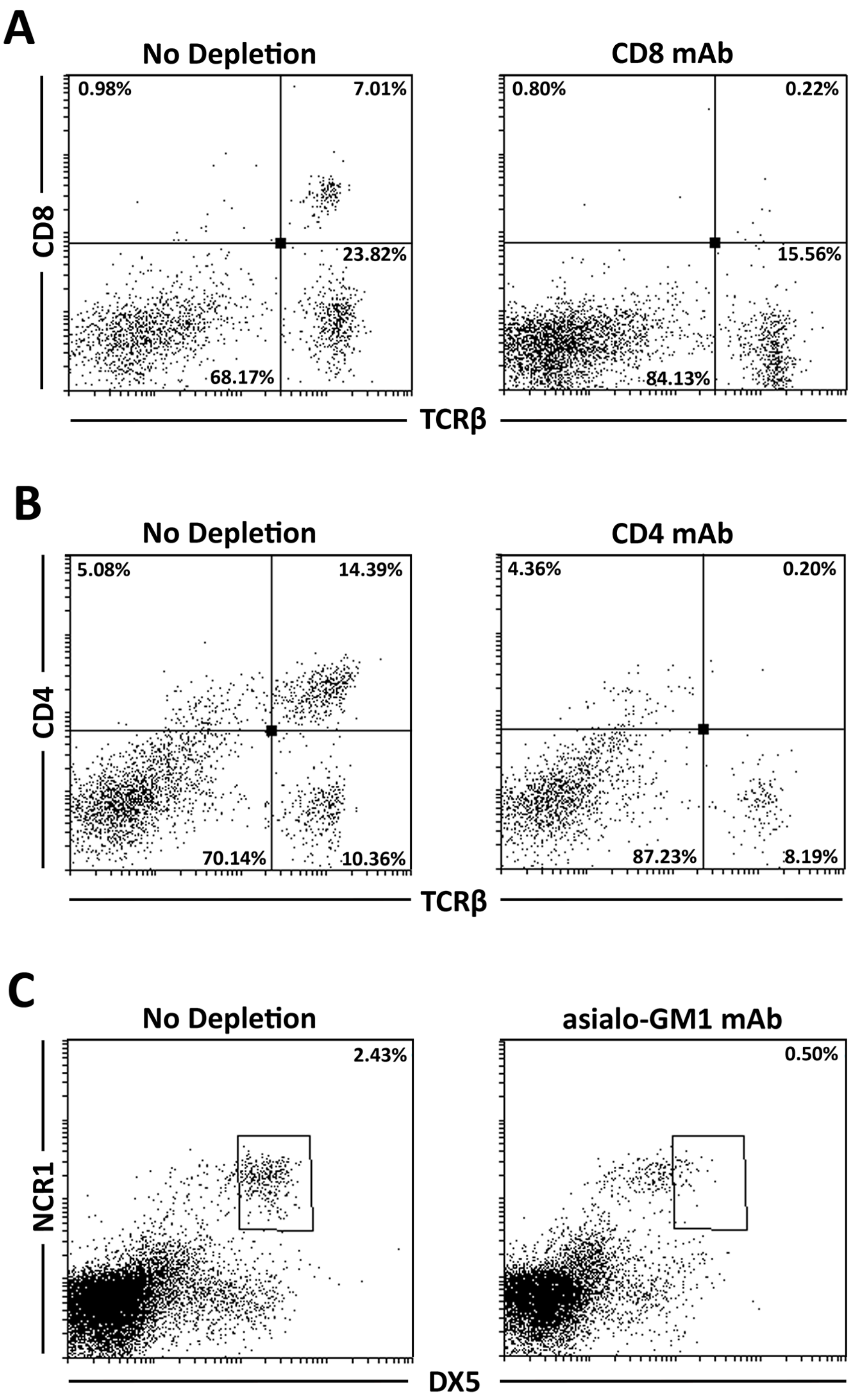

Supplementary Figure 3: CD8+ T cells, CD4+ T cells and NK cells are depleted with mAb treatment. **(A-C)** Representative flow cytometry dot plots of splenocytes harvested from mice with (right column) or without (left column) mAb depletion. (A) Splenocytes were stained with CD8 and TCRb. CD8 mAb depletion reduced CD8+, TCRb+ from 7.01% to 0.22%. (B) Splenocytes were stained with CD4 and TCRb. CD4 mAb depletion reduced CD4+, TCRb+ from 14.39% to 0.20%. (C) Splenocytes were stained with NCR1 and DX5. Asialo-GM1 mAb depletion reduced NCR1+, DX5+ from 2.43% to 0.50%.

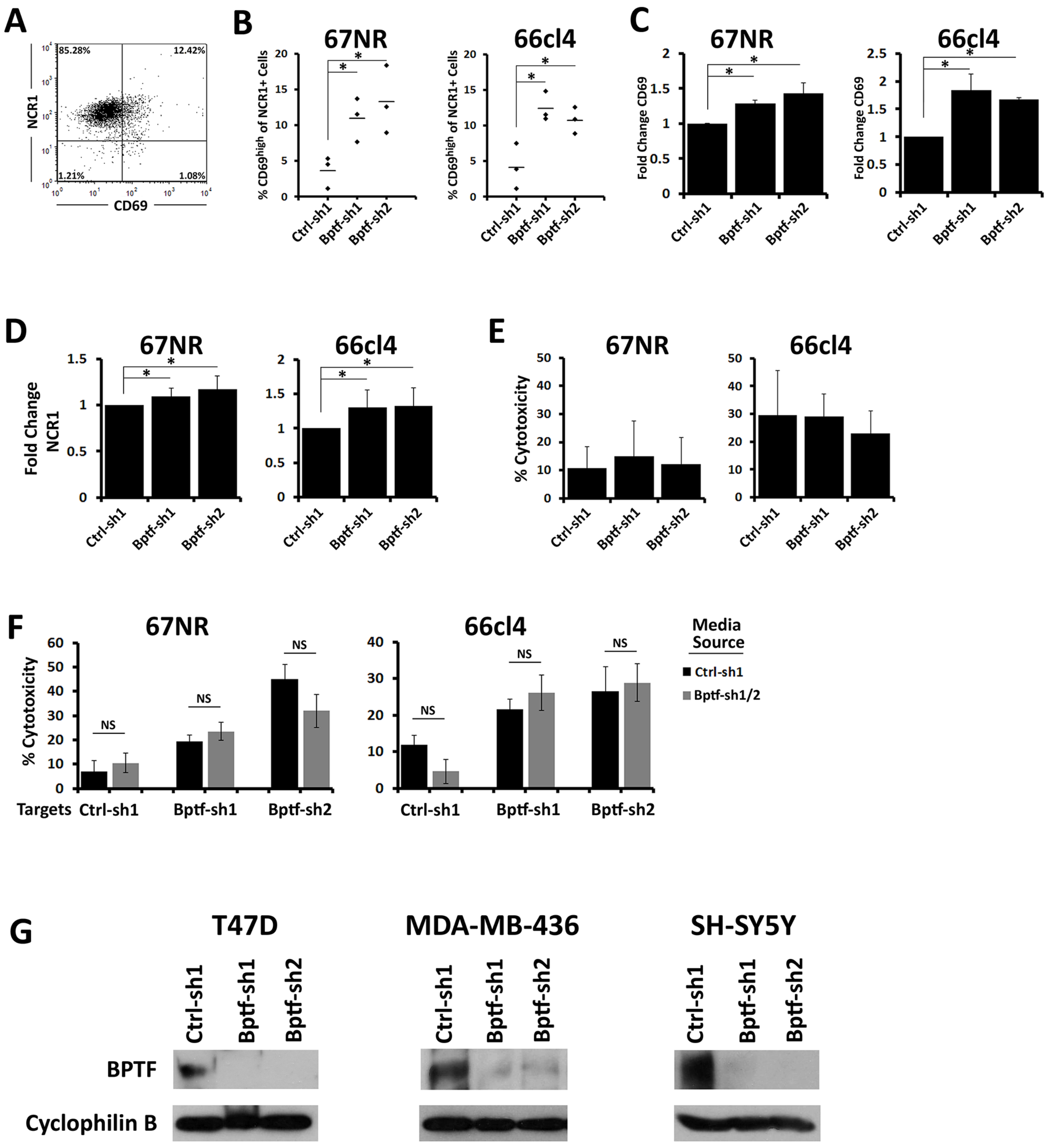

Supplementary Figure 4: A cell surface factor enhances NK cell activation to BPTF depleted tumor cells. (A) Representative flow cytometry dot plot and percentages of live mouse NK cells stained with NCR1 and CD69 after coculture with 66cl4 or 67NR targets at a 5:1 E:T ratio. **(B)** Scatter plot analysis of % CD69high NCR1+ cells from A (n = 3 biological replicates, * = ttest pvalue < 0.04). **(C)** Fold change of CD69 MFI on mouse NK cells from A (n ≥ 4 biological replicates, * = ttest pvalue < 0.01). **(D)** Fold change of NCR1 MFI on mouse NK cells from A (n ≥ 4 biological replicates, * = ttest pvalue < 0.05). **(E)** Cytolytic activity of mouse NK cells treated with PMA + Ionomycin and cocultured with 67NR and 66cl4 targets at a 5:1 E:T ratio (n = 3 biological replicates). **(F)** Mouse NK cells were preincubated with either fresh media or media harvested from control or BPTF KD tumor cells before assay on control or BPTF KD targets at a 5:1 E:T ratio (n = 3 biological replicates, NS = not significant). **(G)** BPTF Western blot analysis of control and BPTF KD T47D, MDA-MB-436 and SH-SY5Y total cell extracts. All quantitative data shown represents mean ± stdev.

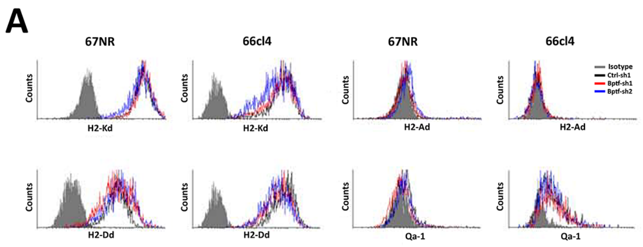

B

67NR NSG

	Gene Count	Benjamini
GO Term: Biological Process		
Cell cycle phase	20	$2.3x10^{-2}$
Mitotic cell cycle	17	$1.2x10^{-2}$
Cell cycle process	22	$9.1x10^{-3}$
M phase	18	$1.0x10^{-2}$
M phase of mitotic cell cycle	14	$2.4x10^{-2}$
Regulation of cell proliferation	25	$2.1x10^{-2}$
Cell cycle	27	$2.0x10^{-2}$
Nuclear division	13	$4.8x10^{-2}$
Mitosis	13	$4.8x10^{-2}$
Vasculature development	15	$4.7x10^{-2}$

66cl4 NSG

	Gene Count	Benjamini
GO Term: Cellular Component		
Plasma membrane part	95	$2.1x10^{-2}$
Cell projection part	19	$2.2x10^{-2}$
Plasma membrane	149	$1.9x10^{-2}$
Cell projection	41	$2.6x10^{-2}$

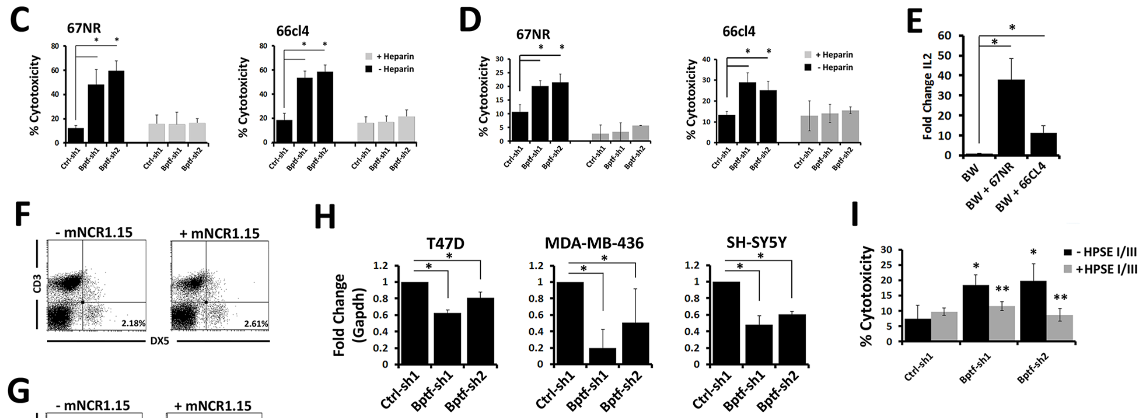

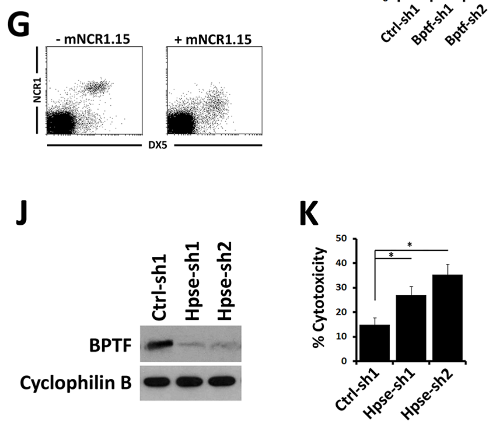

L

Gene Name	HSPG Core Protein	67NR Fold Chage	66cl4 Fold Change
Sdc1	Syndecan-1	1.1	1.2
Sdc2	Syndecan-2	1.6	-1.0
Sdc3	Syndecan-3	1.1	1.1
Sdc4	Syndecan-4	-1.1	-1.1
Gpc1	Glypican-1	1.4	-1.0
Gpc2	Glypican-2	NA	NA
Gpc3	Glypican-3	1.2	1.1
Gpc4	Glypican-4	-1.4	-1.1
Gpc5	Glypican-5	NA	NA
Gpc6	Glypican-6	1	1.1
Tgfbr3	Betaglycan	1	1.1

Gene Name	Sheddase	67NR Fold Chage	66cl4 Fold Change
MMP7	matrix metalloproteinase 7	1	1.2
MMP2	matrix metalloproteinase 2	3	1.1
MMP9	matrix metalloproteinase 9	1.1	-1.3
MMP16	matrix metalloproteinase 16	NA	1.1
ADAM17	A disintegrin and metalloprotease 17	-1.1	-1.1
ADAMTS1	A disintegrin-like and metalloproteinase with thrombospondin motifs-1	1.4	-1.5
ADAMTS4	A disintegrin-like and metalloproteinase with thrombospondin motifs-4	1	1.0
Plg	Plasminogen	NA	-1.0
Notum	Notum	-1.1	1.3
Psenen	Presenilin	1	1.1

= Significant by NSG Microarray

Supplementary Figure 5: BPTF depletion enhances *Hpse*, and not HSPG core protein, expression. **(A)** Representative flow cytometry histograms of MHC molecules from control and BPTF KD 67NR and 66cl4 cells. **(B)** Microarray GO analysis of BPTF-dependent genes identified by microarray gene expression profiling on 67NR and 66cl4 tumors from NSG mice. **(C)** and **(D)** Percent target cell cytotoxicity as measured by LDH assay. (C) NK-92 cells or (D) mouse NK cells were cocultured on 66cl4 or 67NR targets at a 5:1 E:T ratio with or without heparin (n = 3 biological replicates, * = ttest pvalue < 0.05). **(E)** qRT-PCR analysis of *Il-2* expression by NCR1-ζ Il2 reporter BW cells after coculture with 67NR or 66cl4 cells (n = 3 biological replicates, * = ttest pvalue < 0.05). **(F)** and **(G)** Representative flow cytometry dot plots of splenocytes harvested from -/+ mNCR1.15 treated mice. Percentages of CD3- DX5+ NK cells are indicated. **(H)** qRT-PCR analysis of *HPSE* expression in T47D, MDA-MB-436 and SH-SY5Y cells (n = 3 biological replicates, * = ttest pvalue < 0.05). **(I)** Percent cytotoxicity. 66cl4 cells pretreated with bacterial heparinase I/III were cocultured with mouse NK cells at a 5:1 E:T ratio (n = 3 biological replicates, * = ttest pvalue < 0.03)(* = significant to no mAb Ctrl-sh1, ** = significant to the respective no mAb hairpin). **(J)** Western analysis of 66cl4 HPSE KD cell lines. Cyclophilin B was used as a loading control. **(K)** NK cells from naive BALB/c mice were cocultured with control or HPSE KD 66cl4 targets at a 5:1 effector:target (E:T) ratio (n = 3 biological replicates, * = ttest pvalue < 0.05). **(L)** Fold change of HSPG core proteins and sheddases from microarray datasets obtained from 67NR and 66cl4 tumors from NSG mice. All quantitative data shown represent mean ± stdev.

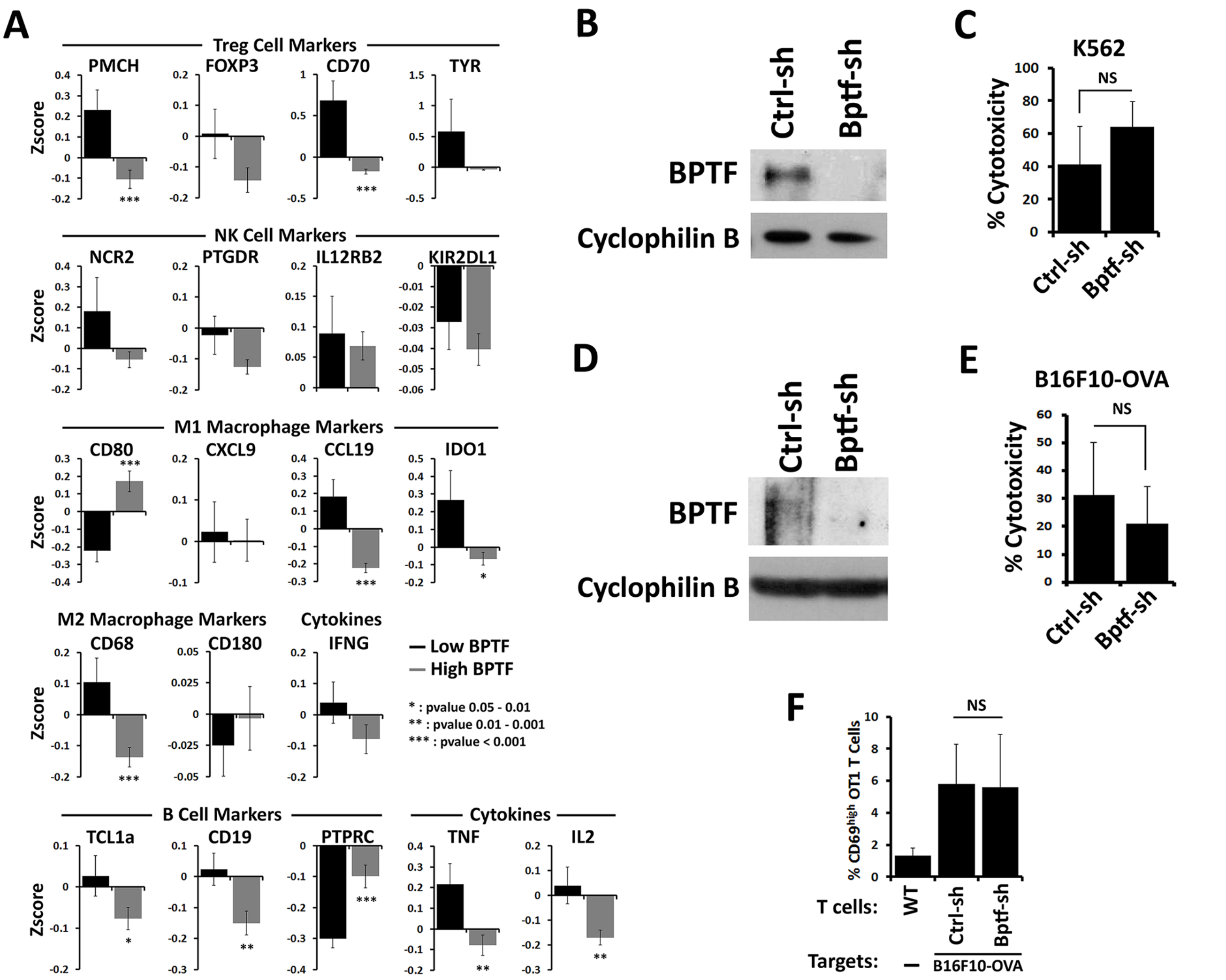

Supplementary Figure 6: BPTF is not required for NK or CD8+ T cell cytolytic activity. **(A)** Differential expression of immune cell markers in *BPTF* high and *BPTF* low expression groups from Zscore normalized expression data obtained from the breast cancer TCGA datasets (pvalues are shown in panel). Data shown represent mean ± sem. **(B)** and **(D)** BPTF Western blot analysis on total cell extracts from control and BPTF KD (B) NK-92 or (D) mouse OT1 T cells after rAd infection. **(C)** and **(E)** Percent target cell cytotoxicity. (C) NK-92 or (E) mouse OT1 T cells were incubated with K562 cells for 4 hrs or B16F10-OVA targets for 24 hrs, respectively, at a 10:1 E:T ratio (n = 3 biological replicates, NS = not significant). **(F)** Percent CD69^{high} of CD8+ OT1 T cells cocultured with or without B16F10-OVA targets. (WT = wildtype, Ctrl-sh = control shRNA, Bptf-sh = Bptf KD shRNA), (n ≥ 3 biological replicates, NS = not significant). All quantitative data shown represent mean ± stdev.

Supplementary Data Set 1: BPTF-dependent genes by microarray and KEGG analysis.

See Supplementary File 1

Supplementary Data Set 2: Primer sequences used in the study.

See Supplementary File 2
